# Implementation of the FilmArray ME panel in laboratory routine using a simple sample selection strategy for diagnosis of meningitis and encephalitis

**DOI:** 10.1186/s12879-020-4904-4

**Published:** 2020-02-22

**Authors:** Susanne Pfefferle, Martin Christner, Martin Aepfelbacher, Marc Lütgehetmann, Holger Rohde

**Affiliations:** Institut für Medizinische Mikrobiologie, Virologie und Hygiene, Universitätsklinikum Hamburg-Eppendorf, Martinistraße 52, 20246 Hamburg, Germany

**Keywords:** Film Array ME, Rapid diagnostic, Syndromic panel testing, Infectious meningitis

## Abstract

**Background:**

Infectious meningitis is a serious disease and patient outcome relies on fast and reliable diagnostics. A syndromic panel testing approach like the FilmArray ME can accelerate diagnosis and therefore decrease the time to pathogen specific therapy. Yet, its clinical utility is controversial, mainly because of a remaining uncertainty in correct interpretation of results, limited data on its performance on clinical specimens and its relatively high costs. The aim of this study was to analyze clinical performance of the assay in a real life setting at a tertiary university hospital using a pragmatic and simple sample selection strategy to reduce the overall cost burden.

**Methods:**

Over a period of 18 months we received 4623 CSF samples (2338 hospitalizations, 1601 individuals). FilmArray ME analysis was restricted to CSF-samples with a high pretest probability of infectious meningitis, e.g. positive Gram-stain, samples in which leukocytes and/or bacteria were evident or urgent suspicion of infection was communicated by clinicians. *N* = 171 samples matched to our risk criteria and were subjected to FilmArray ME analysis. Those samples were also analyzed by reference methods: culture only (*n* = 45), PCR only (*n* = 20) or both methods (*n* = 106).

**Results:**

56/171 (32.75%) were FilmArray ME positive. Bacterial pathogens were detected in 30/56 (53.57%), viral pathogens were detected in 27/56 (48.21%) and yeast DNA was detected in 1/56 (1.79%) of positive samples. Double detection occurred in 2/56 samples. In 52/56 (92.86%) FilmArray ME positive samples, results could be confirmed by the reference assays (sensitivity = 96.30%, specificity =96.58%).

**Conclusion:**

The FilmArray ME assay is a fast and reliable diagnostic tool for the management of infectious meningitis and can easily be implemented in routine diagnostic workflows. However, correlation of test results and underlying clinical symptoms requires experienced users and the awareness of potentially false negative or false positive results. Moreover, considering the need for antimicrobial susceptibility testing, the use of molecular tests as a stand-alone diagnostic cannot be recommended.

## Background

Infectious meningitis is a serious threat with high morbidity and mortality rates [1–4]. In bacterial meningitis, patient outcome can be optimized through immediate antibiotic treatment, thus fast and reliable diagnostic can be crucial [2, 5]. Direct identification of viral, bacterial and fungal pathogens in cerebrospinal fluid (CSF) by molecular testing methods may substantially decrease the time to pathogen specific therapy. However, CSF is a delicate clinical sample, limiting the diagnostic possibilities by its low quantity.

The Biofire FilmArray ME Panel provides for a simultaneous multiplex testing of a comprehensive panel of 14 pathogens using a minimal amount of CSF (e.g. 200 μl). This sample-to-answer system was FDA-cleared in 2015. However, in the multicenter evaluation study, reported detection rates for bacterial pathogens typically found in infectious meningitis cases were surprisingly low. Most notably, *N. meningitides*, a worldwide leading meningitis pathogen, and *L. monocytogenes* detected in none of the samples [6]. Meanwhile, clinical data on the performance of the FilmArray ME was reported by various users worldwide [7–13] with diagnostic specificity and sensitivity in good concordance to the multicenter study Admittedly, both false positive and false negative detections have been reported throughout the studies, with one example of an erroneously diagnosed HSV-1 meningitis in a patient with an underlying tuberculous meningitis [14] and one study indicating a potential of missing viral infections in a pediatric cohort [10]. Aside from the inarguably higher costs of multiplex molecular detection methods compared to standard laboratory procedures like bacterial culture, the remaining uncertainty in interpretation of results is a key point in the controversial discussion concerning the clinical utility of such syndromic testing approaches in infectious meningitis/encephalitis [15]. Nevertheless, there is agreement regarding the substantially decreased time-to-result exhibiting a potential benefit for patient outcome whenever involving an adaptation of antibiotic treatment. Yet, studies on prospective clinical specimens indicating the right patient population or determining the ideal testing approach are missing [15]. We report our real-life experience of the implementation of the FilmArray ME panel in addition to available conventional cultural and molecular diagnostics from CSF in daily laboratory routine in a university hospital setting. In order to avoid unnecessary and cost intensive rapid diagnostics, FilmArray ME analysis was restricted to CSF-samples with a high pretest probability of infectious meningitis. Risk for infection was prospectively assessed by Gram-stain, and only samples in which leukocytes and/or bacteria were evident or urgent suspicion of infection was communicated by clinicians were subsequently analyzed with the Film Array ME assay.

## Methods

### Clinical specimens and selection strategy

**Fig. 1:**
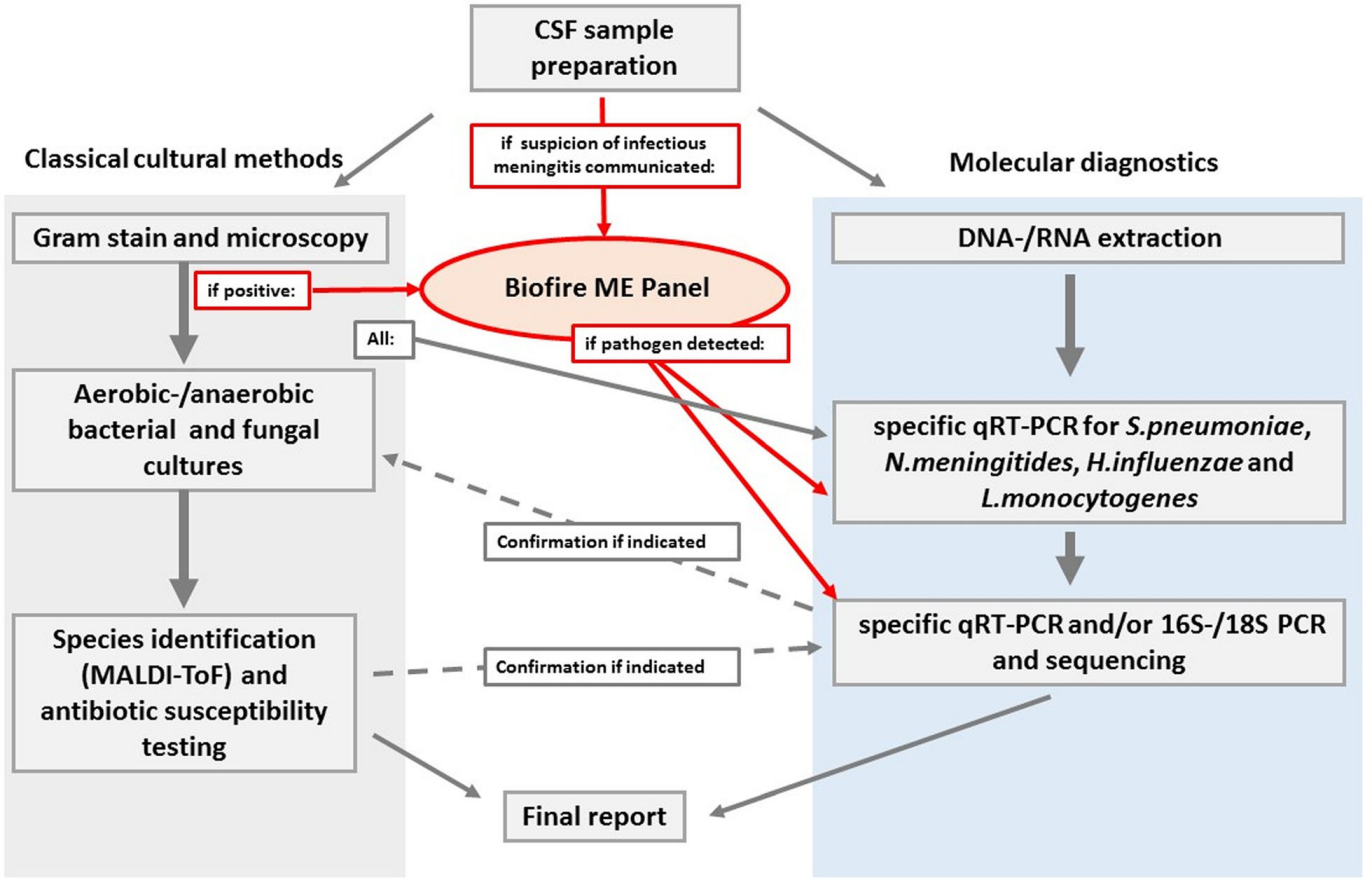
Laboratory workflow: CSF sample will either be analyzed by classical cultural methods (left, gray) and/or molecular methods (right, blue) depending on parameters requested by sender. If suspicion of infectious meningitis is communicated or gram stain is suspect, additional Biofire ME panel will be performed (red). Results will be confirmed by methods applicable for pathogen detected (dashed arrows)

The study was conducted over a period of approximately 18 months (September 2015 through February 2017) in our laboratory. CSF samples demonstrating abnormality in Gram-stain (e.g. leucocytes and/or bacteria visible) and CSF samples of patients with urgent suspicion of infection as communicated by clinicians were selected for additional FilmArray ME analysis (see Fig. 1) Gram stain abnormalities were chosen for ensuring the most rapid decision-making. The FilmArray ME panel could not be ordered directly by clinicians, yet results were reported.

### Ethics

This work was conducted in accordance with §12 of the Hamburg hospital law (§12 HmbKHG).

### Primary bacterial culture and gram-stain

CSF samples were centrifuged for 10 min at 3000 rpm at room temperature. Supernatant was used for *Bacillus subtilis* inhibition-assay for preliminary assessment of presence of antibiotics [16]. With one drop of sediment, a smear was generated on glass slides. Gram-staining was performed after drying and fixation of smear. Equal amounts of sediment were plated onto blood agar plates, chocolate agar plates and Sabouraud agar plates and incubated for 48 h at 35–37 °C with 5% CO_2_, one drop of CSF sediment was inoculated in thioglycollate medium (thioglycollate broth) and incubated at 37 °C. All Plates and broth are inspected at least once daily for bacterial growth. Species identification relied on MALDI-TOF technique combined with classical biochemical identification methods.

### NAAT analysis

Nucleic acid extraction (RNA and DNA) was performed on a QIAsymphony SP/AS instrument with the QIAsymphony DSP Virus/ Pathogen Mini kit using 200 μl of native CSF sample. During extraction process, universal PCR inhibition control (DNA/RNA) was added. Elution volume was 60 μl. Quantitative real-time-PCR reaction was set-up using 5–10 μl of eluate, reverse-transcription, amplification and analysis were performed on a LightCycler 480 instrument. The quantitative real-time PCRs used in CSF diagnostic are laboratory developed tests (*LDTs*), specific primers and probes are designed based on sequences and assays published elsewhere [17–31]. Table 1 gives a list of LDTs used for detection of pathogens included in the FilmArray ME with underlying references. For specific detection of human herpesvirus 1 and human herpesvirus 2, commercially available RealStar® PCR Kit (ATD) was used for verification of results. For molecular detection of BKV, JCV and EBV (human herpesvirus 4) in CSF samples, also molecular LDTs were used [23, 28, 29]. For universal molecular detection of bacterial 16S rRNA and mycotic 18S rRNA, SepsiTest™-UMD (Molzym) was used according to the manufacturer’s instructions. Briefly, 1000 μl of CSF were used for nucleic acid extraction, amplification and melting curve analysis was performed on a LightCycler® 2.0 instrument.. The sequence of amplicons was determined using Sanger sequencing. Sequences were analyzed with SepsiTest BLAST (*www.sepsitest-blast.de/en/index.html*) and the BLAST tool of NCBI (*www.ncbi.nlm.nih.gov/blast**).*

**Table 1:** Laboratory procedures for each pathogen included in the Biofire FilmArray ME panel are listed (X = performed regularly). Primary culture included cultivation of CSF samples at 35–37 °C with 5% CO_2_, all samples were plated on blood agar plates (BAB), chocolate agar (CHOC) and sabouraud agar, and were incubated in fluid thioglycollate medium (thioglycollate broth), respectively. In case of CSF-samples sent for detection of yeast and bacterial pathogens, gram stain and primary culture are performed routinely, NAT analysis is performed if mandatory (e.g. verification of bacteria seen in gram stain). Additionally, bacterial 16S rRNA genes and mycotic 18S rRNA sequences are detected via commercially available SepsiTest™-UMD (Molzym) upon request. For all viral pathogens, primarily quantitative real-time PCR was performed. In these samples, gram stain was only performed upon request (*^1^). For *Human herpesvirus 1* and *human herpesvirus 2*, commercially available RealStar®-PCRs (Altona Diagnostics) were performed for confirmation if indicated. Diagnostic PCRs for CSF diagnostic in our laboratory are laboratory developed tests (“LDTs”), all LDTs are quantitative real-time PCRs using specific primers and probes. Design of LDTs is based on references given into parentheses

	Pathogen	Gram stain	Primary culture	NAT
Bacteria	*Escherichia coli K1*	X	X	LDT (Diaz, Waller [21]/ SepsiTest™-UMD
	*Haemophilus influenzae*	X	X	LDT (Abdeldaim, Stralin [18])/SepsiTest™-UMD
	*Listeria monocytogenes*	X	X	LDT (Le Monnier, Abachin [25])/SepsiTest™-UMD
	*Neisseria meningitidis*	X	X	LDT (Abdeldaim, Stralin [18])/SepsiTest™-UMD
	*Streptococcus agalactiae*	X	X	LDT (Diaz, Waller [21])/SepsiTest™-UMD
	*Streptococcus pneumoniae*	X	X	LDT (Stralin, Herrmann [30])/SepsiTest™-UMD
Viruses	*Cytomegalovirus*	X*^1^		LDT (Khansarinejad, Soleimanjahi [24])
	*Enterovirus*	X*^1^		LDT (Dierssen, Rehren [22])
	*Herpes simplex virus 1*	X*^1^		LDT (Meylan, Robert [26])/RealStar® HSV
	*Herpes simplex virus 2*	X*^1^		LDT (Meylan, Robert [26])/RealStar® HSV
	*Human herpesvirus 6*	X*^1^		LDT (Cassina, Russo [20])
	*Human parechovirus*	X*^1^		LDT (Benschop, Molenkamp [19])
	*Varizella zoster virus*	X*^1^		LDT (Pollak, Dovrat [27])
Yeast	*Cryptococus neoformans/gattii*	X	X	LDT (Veron, Simon [31])/SepsiTest™-UMD

### FilmArray ME panel

FilmArray ME Panel testing was performed according to the manufacturer’s instructions. Briefly, 200 μl of CSF specimen were mixed with sample buffer by inverting. Sediment leftover or supernatant was used if samples were entirely processed for primary culture (off-label use). Hydration solution was injected into the FilmArray ME pouch to rehydrate the freeze-dried reagents, sample mixture was thereafter injected. The pouch was transferred to the instrument and run was initiated. Nucleic acid purification, reverse transcription, multiplex PCR and melting curve analysis are performed automatically, with a runtime of about 1 h. Test reports are generated by instrument software.

## Results

Over a period of 18 months we received a total of *n* = 4623 CSF samples (2338 hospitalizations, 1601 individuals), including samples sent for both molecular diagnostic and bacterial culture as well as samples sent only for either purpose. In detail, bacterial culture was performed in *n* = 2682 samples, molecular diagnostic was performed in *n* = 1891 samples and *n* = 50 samples were sent for serology purpose. The overall positivity rate was 5.97% with positive results in 137/2682 samples analyzed by culture (5.11%) and 139/1891 positive results in samples analyzed by molecular methods (7.35%). More details are given in supplementary Tables 1 and 2.

Overall, *n* = 171 samples matched to our risk criteria and were subjected to FilmArray ME analysis. Of those, *n* = 117 samples had abnormality in gram-stain (*n* = 116 samples with leukocytes, one samples with bacteria seen without evidence for leukocytes) and *n* = 54 samples were selected as suspicion of infectious meningitis was communicated by clinicians. Of note, in 24/54(44.44%) of samples selected upon clinical request, gram stain and primary bacterial culture was not performed as suspicion for viral infection was communicated. 6/30 samples of that subset had positive gram stain (20.00%), whereas 25/30 samples had negative gram stain (83.33%). Overall, gram stain was performed in 147/171 samples. Leucocytes were seen in 122/147 (82.99%) samples, bacteria in 15/147 (10.20%) samples and yeast in 1/147 (0.68%) of those samples.

**Fig. 2:**
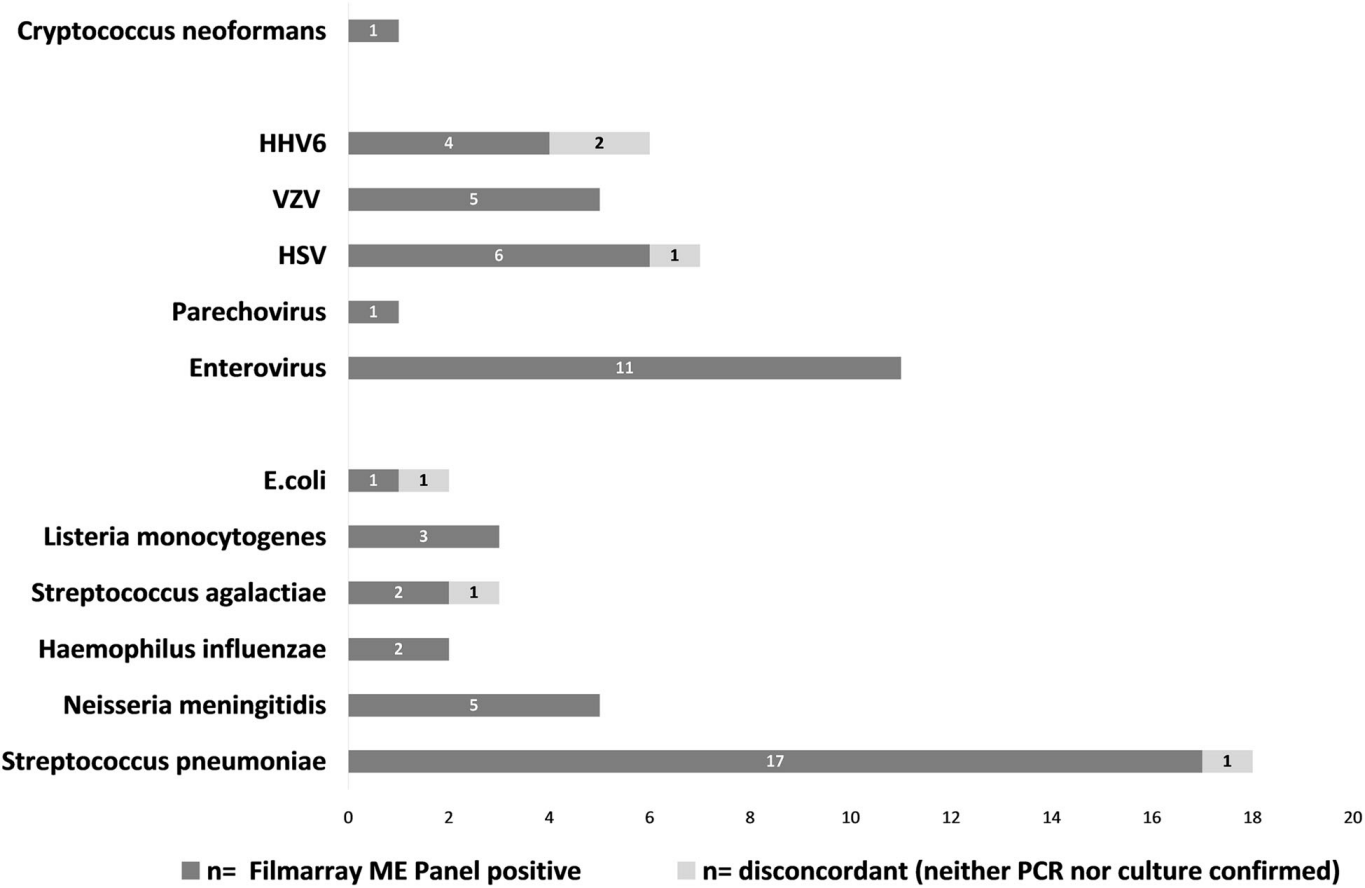
Summary of FilmArray ME positive result (dark gray). Light gray bars indicate disconcordant results, e.g. positive FilmArray ME results that could neither be confirmed by standard cultural methods nor by molecular testing. For *S. pneumoniae*, one sample could not be confirmed by specific PCR nor by cultural methods, whereas *n* = 16 samples were confirmed by specific PCR and *n* = 12 of FilmArray ME positive samples were confirmed by both methods. For *S. agalactiae*, one of the FilmArray ME positive samples could neither be confirmed by cultural methods nor by specific PCR. For *E.coli K1* detected in FilmArray ME also no confirmation was obtained by any method. One sample was designated *HSV-1* positive by FilmArray ME, but no such pathogen could be detected by two different specific quantitative real-time PCRs. *HHV-6* was detected in four samples by Film Array ME, two of those yielded a double hit (positive for *HHV-6* and *VZV or H. influenzae and HHV-6, respectively*). Conventional assays confirmed presence of *VZV* and *H. influenzae*, whereas *HHV-6* was not found by specific PCR

Samples analyzed by FilmArray ME were also tested by reference methods: culture only (*n* = 45), molecular analysis only (*n* = 20) or both methods (*n* = 106). 56/171 (32.75%) were FilmArray ME positive. Bacterial pathogens were detected in 30/56 (53.57%) and viral pathogens were detected in 27/56 (48.21%) of positive samples. Additionally, *Cryptococcus neoformans* was detected in 1/56 samples (1.79%). Double detection occurred in two samples. An overview of pathogens detected is given in Fig. 2.

Within the subset of samples selected for leucocytes seen in gram stain (*n* = 116), positive Film Array ME results were obtained in 44/116 samples (positivity rate 37.93%, sensitivity = 99.45%, specificity = 10.96%). Organisms have been seen in 16/117 samples selected for positive gram stain (bacteria in 15/117 samples (12.82%), yeast in 1/117 samples (0.85%, positivity rate = 13.63%, sensitivity = 22.73%, specificity = 91.78%). Within the subset of samples selected upon request by clinicians, (*n* = 54), positive Film Array ME results were obtained in 44/54 samples (positivity rate = 22.22%, sensitivity = 91.67%, specificity = 97.62%).

### Clinical performance

52/56 positive FilmArray ME results could be confirmed by the reference assays (sensitivity = 96.30%, specificity = 96.58%, PPV = 92.86%). In four samples that were positive for *S. pneumoniae, E. coli, S. agalactiae or HSV-1* in FilmArray ME panel, the result could not be confirmed by a reference method (Fig. 2, light grey bars). For the three samples positive for bacterial pathogens *E.coli, S. pneumonia* and *S. agalactiae*, primary culture showed no bacterial growth after incubation period. Specific real-time PCR was performed in the two samples positive for *S. pneumoniae* and *S. agalactiae*, but no signal was obtained. Also, no bacterial 16S-rRNA was detected in the sample positive for *S. pneumoniae* in SepsiTest®-UMD (Molzym). Residual material of the two samples positive for *E. coli K1* and *S. agalactiae* was not sufficient for additional 16S-rRNA amplification, as proper DNA extraction for this method requires a high input volume (e.g. 1000 μl of CNS liquid). No specific real-time PCR assay for detection of *E.coli K1* was applied. One sample that was FilmArray positive for HSV-1 could not be confirmed by two independent molecular detection methods. Neither our laboratory developed real-time PCR assay, nor a commercially available real-time PCR assay yielded a positive result (see Table 1 for assay details).

Two samples (1%) revealed negative FilmArray results, but were positive for *S. pneumoniae* and *Parechovirus* by specific in-house PCRs, respectively (NPV =98.26%). In two samples, FilmArray analysis detected two pathogens (*VZV* and *HHV-6*, and *H. influenzae* and *HHV-6*, respectively). Presence of *VZV* (1.4 × 10^4^ copies/ml) and *H. influenzae* was confirmed by specific real-time PCRs, but conventional assays were unable to detect *HHV-6*, and these results were thus regarded as false-positive. In 10 samples, pathogens not found by the FilmArray ME panel were detected by culture (*Bacillus sp*., CoNS, *S. aureus*, *K. pneumoniae*) or PCR (BK virus, *Streptococcus spp*.).

### Estimation of the impact of our risk-assessment driven sample selection strategy

**Fig. 3:**
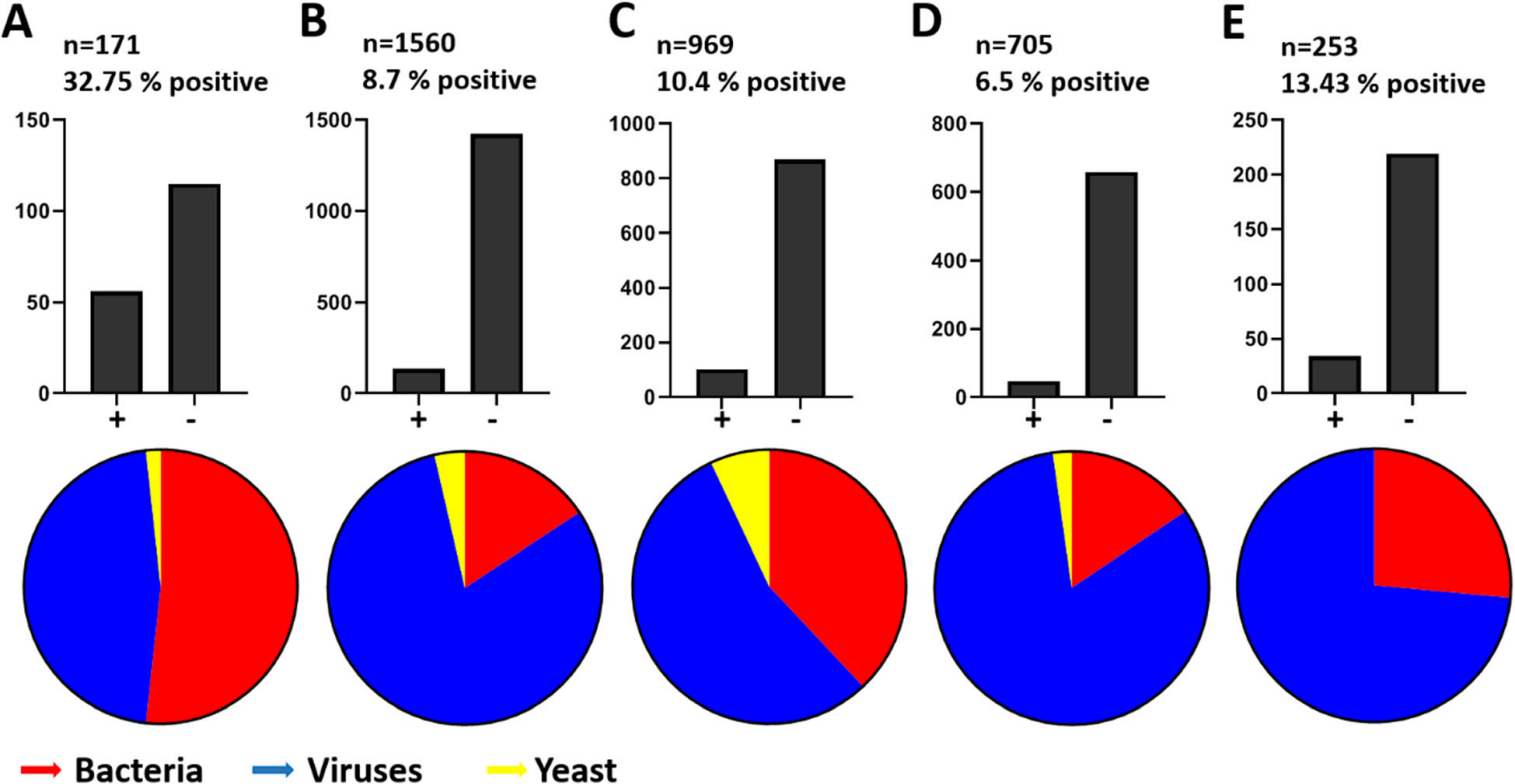
Comparison of Film Array ME positivity rates and pathogen composition in different studies. Our study (**a**) is compared to the multicenter evaluation study (**b**) and three studies with high numbers of samples tested (**c**-**e**). Bar diagrams reflect numbers of positive (left bar) and negative (right bar) samples. Pie charts illustrate pathogen composition within positive samples. **a** This study analyzed *n* = 171 CSF specimens (of 4623 received) by Film Array ME, the overall positivity rate was 32.75% with bacteria (red) detected in 53.57% of positive samples, followed by viruses (blue) detected in 48.21% of positive samples and yeast (yellow) detected in 1.79% of positive samples. **b** In the multicenter evaluation study [6] *n* = 1560 samples were analyzed by Film Array ME and the overall positivity rate was 8.7%. Bacteria were detected in 16.2% of positive samples, viruses in 83.8% of positive samples and yeast in 3.7% of positive samples. **c** Tarai et al. [7] reported a positivity rate of 10.4% in *n* = 969 CSF samples analyzed. In their study, bacteria were detected in 37.2%, viruses in 54.46% and yeast in 6.93% of positive samples, respectively. **d** Radmard et al. reported a retrospective review of *n* = 705 CSF specimens tested with an overall positivity rate of 6.5%. In that study, bacteria were detected in 15.21%, viruses in 80.43% and yeast in 2.17% of positive samples, respectively. **e** In a prospective study monitoring unrestricted physician ordered testing, Naccache et al. observed an overall positivity rate of 13.43% of *n* = 253 samples tested, with bacterial pathogens in 26.47% and viral pathogens in 73.52%. No yeast were found in that study

Results were compared to the multicenter evaluation study [6] and three other studies [7, 32, 33], that reported Film Array performance data on a high quantity of CSF samples tested (*n* = 1560, *n* = 969, *n* = 705 and *n* = 253 CSF samples analyzed by Film Array ME, respectively). Figure 3 illustrates positivity rates and pathogen composition in CSF samples analyzed by different studies.

## Discussion

We here report on our real-life experience of the implementation of the FilmArray ME panel into a diagnostic laboratory in a German university hospital setting. We implemented a simple sample selection strategy in our workflow: FilmArray ME analysis was restricted to CSF-samples with a high pretest probability of infectious (bacterial) meningitis. Decision was made in our laboratory based on gram stain and/or urgent suspicion of infectious meningitis communicated by clinicians, e.g. neither the clinical decision for lumbar puncture was affected, nor was explicit ordering of the assay permissible. Using this selection criteria we selected 171/4623 CSF specimens for additional syndromic molecular testing. Overall we observed positive results in 56/171 (32%), which is higher than in other published studies [6, 7, 9–12, 34–38] and substantially higher compared to the multicenter evaluation study [6] and three other studies [7, 32, 33], that reported positivity rates of under 15% (*p* < 0.0001). In addition, we could increase the amount of bacterial pathogens to 53% of the specimens, again this is more than in other published studies that observed positive ranges for bacterial pathogens between 15 and 37% [6, 7, 32, 33]. Indeed, bacteria were the most abundant pathogens detected in our study (53.57% of positive samples), followed by viruses detected in 48.21% of positive samples. In contrast, in the comparator studies viruses were detected most frequently (83.8 -54.46% of positive samples) followed by bacteria (37.62 - 15.21% of positive samples, *p* < 0.0001).

In concordance with other studies [6, 7, 9–12, 34–38], *S. pneumoniae* (17/30) was the most frequent bacterial pathogen detected by the FilmArray ME assay. Additionally, in 10/30 specimens rapid detection of *N. meningitides* (*n* = 5), *H. influenzae* (*n* = 2) or *L. monocytogenes* (*n* = 3) was important for optimal clinical management including rapid implementation of post exposure treatments in contact persons (antibiotic prophylaxis and/or vaccination).

Analytically we observed good concordance (52/56 positive results could be confirmed) between the FilmArray ME panel results and our comparator assays. Only in two of the samples the FilmArray ME panel yielded a discordant negative result compared to the local reference methods. Namely, in each of one sample *S. pneumoniae* and *Parechovirus* were detected by specific in-house PCRs. Although it might be crucial to detect a bacterial or viral meningitis in the individual case, our data confirm the high negative predictive value of the multiplex PCR assay that has been emphasized by others [33].

In four samples, potentially false positive results of FilmArray ME panel occurred, including three samples tested positive for bacterial pathogens by multiplex PCR that remained culture negative. Among the reasons for failure of primary culture is antibiotic administration prior to lumbar puncture [39]. In the three disconcordant samples, bacterial growth inhibitors (such as antibiotics) were found only in the two samples positive for *E. coli* and *S. pneumoniae*. One sample positive for *S. agalactiae* in FilmArray E panel showed no inhibition of growth in *B. subtilis* inhibition assay. Notably, culture negative yet PCR positive infectious meningitis cases caused by *S. agalactiae* have been described previously, underlining the importance of molecular diagnostic for optimization of patient management [40–42]. Otherwise, it cannot be excluded that in our sample, FilmArray ME detection of *S.agalactiae* was indeed false positive and the communication of the test result might have led to a dispensable antibiotic administration.

In our study, one sample remained disconcordant for *HSV-1*. Beside the probable useless administration of Acyclovir following a false positive *HSV-1* result, one should also consider the questionable relevance of *Herpesviridae* detection in CSF in general. As these viruses feature life-long latency after primary infection, subclinical reactivation with replication can be seen in conjunction with different underlying clinical conditions [43, 44]. Thus, in a worst case scenario, *Herpesviridae* detection in multiplex PCR testing might lead to delayed diagnosis of the actual underlying disease, as described by Gomez et al. for a patient suffering from tuberculous meningitis [14].

The herpesvirus *HHV-6* is included in the FilmArray ME panel. This virus exhibits not only latency and also has the potential of chromosomal integration. Therefore, careful interpretation of *HHV-6* detection in FilmArray ME panel is mandatory. The clinical diagnosis should generally not be made by molecular detection of *HHV-6* in CSF alone [45, 46]. As this study focused on the implementation of the assay into the laboratory workflow, no clinical data was analyzed. The relevance of *HHV-6* detection in our study remains unclear in its consequences for patient management and outcome.

An obvious limitation of any syndromic molecular panel testing approach is the limited number of pathogens included into the panel. Even though the FilmArray ME panel includes a broad range of pathogens, overreliance on negative results might be crucial especially in geographical regions with unusual etiologic agents of meningitis this might yield to ineffective testing [7]. In our study, we detected pathogens that are not included in the panel by comparator assays in 10 samples: *Bacillus sp*., CoNS, *S. aureus*, and *K. pneumoniae* grew on culture media, whereas BK virus and *Streptococcus spp*. were detected by PCR. Albeit the questionable clinical relevance of *Bacillus sp., Streptococcus spp*. and CoNS in a CSF sample, detection of *S. aureus*, *K. pneumoniae* and *BK-Virus* may account for serious infections. This underlines, that reasonable and effective CNS diagnostic should combine classical cultural and molecular methods, rather than focus on one test system alone.

It has been proposed recently that implementation of the FilmArray ME Panel in routine diagnostic may help for cost saving in direct antimicrobial utilization and might decrease diagnostic costs even with uncontrolled routine availability of the assay [47, 48].

However, both Naccache et al. and Radmard et al. reported massive overutilization of the test, when no eligibility criteria was implemented [32, 33]. In the latter study, more than one third of samples analyzed were taken from patients without suspicion for infectious meningitis/encephalitis. The authors advice against potential overreliance of test results and suggest restriction strategies within the scope of diagnostic stewardship programs [49]. Corroborate findings were reported by Tan et al., who describe a potential overutilization of the assay in children, but yet point out the benefits of early diagnosis of a viral etiology, especially in terms of antibiotic usage [50].

Eichinger et al. reported their findings of implementation of the FilmArray ME panel as a POCT in children with suspected meningitis in a children hospital [51]. They highlight that though the availability of rapid diagnostic tests enhances administration of specific treatment and thus reduces inadequate usage of antibiotics, a structured approach in clinical implementation of the assay is needed. Taken together, there is agreement that restriction of the assay might be helpful for enhancing its clinical utility. In a point-counterpoint discussion [15] the pros and cons of syndromic testing approaches in CSF specimens in general have been summed up. Despite their contradictory positions on the use of the FilmArray ME panel in diagnostic laboratory routine, the authors agree on the fact that it ensures reduced turn-around time of molecular results and therefore might be beneficial for patient outcome. Yet, the appropriate patient population for testing still has to be identified and more data on the performance of the assay on clinical specimens are needed to evaluate the ideal approach for testing.

Our implementation approach is independent from approval by infectious disease specialists and it does not involve any ordering modifications. The latter opportunities have been proposed for diagnostic stewardship programs [49], however, implementation of such criteria might slow-down the whole workflow. Since one major benefit of molecular multiplex testing is the fast generation of results with high negative predictive values [33], any retardation might also be discussed controversially. Therefore we believe that our restriction approach can be an easy and effective alternative.

### Limitations of the study

We report our real-life experience of the implementation of the FilmArray ME panel into our laboratory workflow, no sample randomization was done. Clinical specimens were tested prospectively, yet our selection strategy might exhibit a selection bias. Notably, sample selection strategy was mainly based on gram-stain abnormalities ensuring on-spot decision making, but not representing the ideal method for leucocyte detection. Furthermore, we did not assess all samples by all comparator assays, in particular for samples that were not selected for FilmArray ME analysis, diagnostic was performed according to orders and no additional assays were performed. Thus, we cannot rule out, that pathogens have been overlooked in some samples. Moreover, no clinical data was analyzed, hence effects on patient outcome or antibiotic utilization in our study remain unclear. Nevertheless we believe that our data gain new insights in how syndromic panel testing may be implemented into laboratory routine and therefore may help to identify the ideal approach to ensure its clinical utility.

## Conclusion

The FilmArray ME is a useful tool for fast and reliable diagnostic of infectious meningitis in a real life diagnostic setting and can be easily implemented in routine diagnostic workflows. Beyond that, our strategy of risk-assessment driven selection of samples might help to avoid unnecessary testing if bacterial pathogens are suspected. However, correlation of test results and underlying clinical symptoms requires experienced users and the awareness of potentially false negative or false positive results. Moreover, considering the need for antimicrobial susceptibility testing, the use of molecular tests as stand-alone diagnostic cannot be recommended.

## Supplementary information

**Additional file 1: Table S1.** Overview of analytical methods performed and detection rates in *n* = 4623 CSF samples received.

**Additional file 2: Table S2.** Overview of pathogens detected by different methods. Numbers of pathogens detected by routine diagnostic procedures, numbers detected by Film Array ME Panel as well as confirmatory results are given.

## Data Availability

Data used and/or analyzed are available from the corresponding author upon reasonable request.

## Abbreviations

BAB: Blood-agar plates
BKV: BK-Polyomavirus
CHOC: Chocolate-agar plates
CSF: Cerebrospinal fluid
HSV: Human herpesvirus
JCV: JC-Polyomavirus
PCR: Polymerase chain reaction
