# Supplementary material for: Implementation of the FilmArray ME panel in laboratory routine using a simple sample selection strategy for diagnosis of meningitis and encephalitis
Source: BMC Infect Dis. 2020 Feb 22;20:170. doi: 10.1186/s12879-020-4904-4 (PMC7036261; doi:10.1186/s12879-020-4904-4)
Supplement: Supplementary file 1 — Additional file 1: Table S1. Overview of analytical methods performed and detection rates in n = 4623 CSF samples received. [file 12879_2020_4904_MOESM1_ESM.docx]

| **Method** |  | **n CSF samples analyzed** | **n CSF samples positive (%)** |
| --- | --- | --- | --- |
| culture |  | 2682 | 137 (5.11) |
|  | bacterial culture | 2517 | 135 (5.36) |
|  | mycobacterial culture | 116 | 0(0) |
|  | blood culture | 28 | 1 (3.57) |
|  | fungal culture | 21 | 1 (4.76) |
| NAT |  | 1891 | 139 (7.35) |
|  |  |  |  |
| Serology |  | 50 |  |
|  | HIV | 25 |  |
|  | other | 25 |  |

Supplementary table 1: Overview of analytical methods performed and detection rates in n=4623 CSF samples received.
