# Supplementary material for: Implementation of the FilmArray ME panel in laboratory routine using a simple sample selection strategy for diagnosis of meningitis and encephalitis
Source: BMC Infect Dis. 2020 Feb 22;20:170. doi: 10.1186/s12879-020-4904-4 (PMC7036261; doi:10.1186/s12879-020-4904-4)
Supplement: Supplementary file 2 — Additional file 2: Table S2. Overview of pathogens detected by different methods. Numbers of pathogens detected by routine diagnostic procedures, numbers detected by Film Array ME Panel as well as confirmatory results are given. [file 12879_2020_4904_MOESM2_ESM.docx]

| **Pathogens detected by NAT** | | **Number of detection** | | | | |
| --- | --- | --- | --- | --- | --- | --- |
|  |  | Routine | FilmArray ME Panel | | Confirmation of FilmArray ME results by NAT | |
| Pathogens included in the FilmArray ME Panel | *Neisseria meningitidis* | 3 | | 5 | 5 | |
|  | *Listeria monocytogenes* | 3 | | 3 | 3 | |
|  | *Haemophilus influenzae* | 1 | | 2 | 2 | |
|  | *E.coli* | 0 | | 1 | 0 | |
|  | *Streptococcus agalactiae* | 0 | | 2 | 1 | |
|  | *Streptococcus pneumoniae* | 8 | | 17 | 16 | |
|  | CMV | 2 | | 0 | nd | |
|  | HSV-1 | 10 | | 2 | 1 | |
|  | HSV-2 | 8 | | 4 | 4 | |
|  | HHV-6 | 5 | | 4 | 2 | |
|  | Enterovirus (including Parechovirus) | 14 | | 12 | 12 | |
|  | VZV | 36 | | 5 | 5 | |
|  | Cryptococcus neoformans | 1 | | 1 | 1 | |
| Pathogens not included in the FilmArray ME Panel | *Klebsiella pneumoniae* | 1 | |  |  | |
|  | *Hydrogenophilus spp* | 1 | |  |  | |
|  | *Streptococcus spp* | 2 | |  |  | |
|  | *Enterobacter spp* | 1 | |  |  | |
|  | CoNS | 5 | |  |  | |
|  | Adenovirus | 1 | |  |  | |
|  | BKV | 1 | |  |  | |
|  | JCV | 3 | |  |  | |
|  | EBV | 29 | |  |  | |
|  |  |  | |  |  | |
|  | *Toxoplasma gondii* | 4 | |  |  | |
| **Pathogens detected by Culture** | | **Number of detection** | | | | |
|  |  | Routine | | FilmArray ME Panel | | Confirmation of FilmArray ME results by culture |
| Pathogens included in the FilmArray ME Panel | *Neisseria meningitidis* | 1 | | 5 | 1 | |
|  | *Listeria monocytogenes* | 3 | | 3 | 3 | |
|  | *Haemophilus influenzae* | 2 | | 2 | 2 | |
|  | *Escherichia coli* | 0 | | 1 | 0 | |
|  | *Streptococcus agalactiae* | 0 | | 2 | 0 | |
|  | *Streptococcus pneumoniae* | 13 | | 17 | 12 | |
|  | *Cryptococcus neoformans* | 1 | | 1 | 1 | |
|  |  |  | |  |  | |
| Pathogens not included in the FilmArray ME Panel | *Acinetobacter lwoffii* | 1 | |  |  | |
|  | *Bacillus species* | 3 | |  |  | |
|  | CoNS | 65 | |  |  | |
|  | *Corynebacterium species* | 3 | |  |  | |
|  | *Enterobacter aerogenes* | 6 | |  |  | |
|  | *Enterobacter cloacae complex* | 3 | |  |  | |
|  | *Enterococcus faecalis* | 1 | |  |  | |
|  | *Enterococcus faecium* | 5 | |  |  | |
|  | *Klebsiella pneumoniae* | 7 | |  |  | |
|  | *Micrococcus species* | 6 | |  |  | |
|  | *Moraxella species* | 3 | |  |  | |
|  | *Pseudomonas aeruginosa* | 2 | |  |  | |
|  | *Staphylococcus aureus* | 4 | |  |  | |
|  | *Streptococcus species* | 4 | |  |  | |
|  | *Aspergillus fumigatus* | 1 | |  |  | |
|  | *Candida albicans* | 3 | |  |  | |

**Supplementary table 2:** Overview of pathogens detected by different methods. Numbers of pathogens detected by routine diagnostic procedures, numbers detected by Film Array ME Panel as well as confirmatory results are given.
